# Glucose Transporter Deficiency Syndrome Type 1 (Glut1‐DS): New Insights From a Brazilian Cohort of Patients

**DOI:** 10.1002/jmd2.70113

**Published:** 2026-08-23

**Authors:** Lívia Maria Ferreira Sobrinho, Juliana Cristine Fontana, Claudia F. Lorea, Helio van der Linden Júnior, Andréa C. S. Leite, Rayana Elias Maia, Lilia Farret, Carolina F. Moura de Souza, Soraia Poloni, Fabiano O. Poswar, Ida Vanessa D. Schwartz, Fernanda Sperb‐Ludwig

**Affiliations:** ^1^ Postgraduate Program in Genetics and Molecular Biology Federal University of Rio Grande do Sul (UFRGS) Porto Alegre Rio Grande do Sul Brazil; ^2^ Department of Pediatrics, School of Medicine Federal University of Minas Gerais Belo Horizonte Minas Gerais Brazil; ^3^ Clinical Research Center Hospital de Clínicas de Porto Alegre Porto Alegre Rio Grande do Sul Brazil; ^4^ Dr. Henrique Santillo Rehabilitation Center Goiânia Goiás Brazil; ^5^ Neuro Kids Clinic Montes Claros Minas Gerais Brazil; ^6^ Department of Pediatrics and Genetics Federal University of Paraíba João Pessoa Paraíba Brazil; ^7^ Medical Genetics Service Hospital de Clínicas de Porto Alegre Porto Alegre Rio Grande do Sul Brazil; ^8^ Casa dos Raros Porto Alegre Rio Grande do Sul Brazil; ^9^ BRAIN Laboratory, Experimental Research Center Hospital de Clínicas de Porto Alegre Porto Alegre Rio Grande do Sul Brazil; ^10^ InRaras–Instituto Nacional de Ciência e Tecnologia Em Doenças Raras Porto Alegre Rio Grande do Sul Brazil

**Keywords:** developmental disabilities, Genetic, GLUT1 deficiency syndrome, ketogenic diet, *SLC2A1*

## Abstract

Glucose transporter deficiency syndrome type 1 (Glut1‐DS) is a rare neurometabolic disorder caused by pathogenic variants in *SLC2A1*, characterized by epilepsy, neurodevelopmental delay, movement disorders, dysarthria, intellectual disability, and postnatal microcephaly. We aimed to characterize the natural history of a Brazilian cohort of patients with Glut1‐DS. Brazilian patients of all ages were included if they presented with clinical features consistent with Glut1‐DS and carried a pathogenic or likely pathogenic *SLC2A1* variant. Forty patients were included (21 males; median age 9.1 years [IQR: 6.0–12.9]; median age at diagnosis 5.0 years [IQR: 3.0–9.9]), of whom 32 had the classical phenotype. The most frequent manifestations at inclusion were seizures (37/40), speech delay (34/40), and movement disorders (29/35). Thirty‐four patients (85%) were receiving ketogenic dietary therapy (27 classical ketogenic diet and 7 modified Atkins diet). Cerebrospinal fluid analysis was performed in 10 patients (27.5%), all of whom had hypoglycorrhachia. Genetic testing identified 31 distinct *SLC2A1* variants, including 15 novel variants. Intrafamilial phenotypic variability was observed in one of four families with recurrent disease, and reduced penetrance was identified in one affected parent. Missense variants were generally associated with milder phenotypes than truncating variants. These findings expand the phenotypic and genotypic spectrum of Glut1‐DS, highlight the high proportion of previously unreported *SLC2A1* variants in the Brazilian population, and reinforce the importance of comprehensive genetic evaluation and counseling for affected families.

## Key Points

- This study reports the first comprehensive Brazilian cohort describing the natural history of Glut1‐DS.
- Fifteen previously unreported *SLC2A1* variants were identified, expanding the known molecular spectrum of the disease.
- Missense variants were generally associated with milder phenotypes, whereas truncating variants were more frequently associated with severe clinical presentations.
- Familial cases, including parental mosaicism, and reduced penetrance, emphasize the importance of systematic family testing and genetic counseling.
- Early recognition and access to molecular diagnosis remain essential to optimize ketogenic dietary treatment and improve long‐term outcomes.

## 1 Introduction

Glucose metabolism is of paramount importance for neuronal activity. Normally, glucose crosses the blood–brain barrier through the glucose transporter member 1 (GLUT1). This transporter is encoded by the solute carrier family 2, member 1 (*SLC2A1*) gene (OMIM*138140). Pathogenic variants in the *SLC2A1* gene cause defects in these brain transporters or altogether prevent their expression, leading to glucose deficiency, and energy deficiency in the brain [1, 2].

The classic manifestations of glucose transporter deficiency syndrome type 1 (Glut1‐DS) are seizures that begin before the age of 2, developmental delay, dysarthria, postnatal microcephaly, and movement disorders. These signs and symptoms occur in about 90% of cases; however, some patients also show nonclassic, atypical symptoms, such as intermittent ataxia, choreoathetosis, dystonia, and hemiplegia. In most cases, the disease is inherited in an autosomal dominant manner (90% are caused by a de novo variant), but autosomal recessive inheritance is possible [3].

The ketogenic diet is highly recommended for the control of seizures and movement disorders and to allow acquisition of neurocognitive abilities in patients with Glut1‐DS [1]. As a rule, it is well tolerated with hardly any side effects [4].

Very little is known about the natural history of Glut1 deficiency syndrome (Glut1‐DS) across different populations worldwide; its true prevalence and incidence remain uncertain, and accurate epidemiological estimates are still scarce in the literature [4]. Coman et al. estimated the prevalence in Queensland, Australia, to be approximately 1:90 000, while in Scandinavia the prevalence was estimated at 1:83 000 [5, 6]. More recently, a prospective population‐based national cohort study conducted in Scotland by Symonds et al. provided an incidence estimate for early childhood‐onset SLC2A1‐related epilepsy of 1:24 300 live births [7]. Registries of patients with this disease can help define epidemiological factors, elucidate the course of the disease, better delineate phenotypes, and improve genotype–phenotype correlations. This paper describes a cohort of 40 Brazilian patients with Glut1‐DS.

## 2 Methods

Ethical approval was obtained from the Research Ethics Committee of Hospital das Clínicas de Porto Alegre, Rio Grande do Sul, Brazil (Certificate of Submission for Ethical Approval: 65118222.5.0000.5327). Patients were included in the cohort if they presented with clinical features consistent with Glut1‐DS and carried a pathogenic or likely pathogenic variant in the *SLC2A1* gene, classified according to ACMG criteria.

Participating centers known to care for patients with Glut1‐DS, as well as the Brazilian Glut1‐DS Association, were invited to contribute. Once informed consent had been obtained, data were collected through teleconsultations (*n* = 32) or in‐person evaluations (*n* = 8), depending on the patient's geographic origin.

Detailed information on family history, abnormal pre‐ and perinatal events, age at onset of seizures, psychomotor development, age at diagnosis, and response to treatment provided were collected and retrospectively reviewed. Patients were also prospectively evaluated using the Glut‐1CSS (Table 1).

**TABLE 1:** Glut1 deficiency syndrome clinical severity score (Glut1‐CSS).

		Score
Ambulation	Normal	0
	Clumsiness	1
	Autonomous ataxic gait	2
	Outdoor assisted ambulation	3
	Indoor assisted ambulation	4
	Wheelchair‐bound	5
Language	Normal	0
	Delayed acquisition	1
	Mild dysarthria (understandable)	2
	Severe dysarthria (only comprehensible to some family members)	3
	Nonverbal communication	4
	Absence of communication	5
Seizure	No	0
	Yes, controlled by antiepileptic drugs	1
	Yes, controlled by Ketogenic diet	1
	Yes, controlled by antiepileptic drugs and Ketogenic diet No control	2 3
Independent gait acquisition	Before 1 year 6 months	0
	Between 1 year 6 months and 2 years of age	1
	After 2 years of age	2
	Never	3
Language acquisition	Before 1 year 6 months	0
	Between 1 year 6 months and 2 years of age	1
	After 2 years of age	2
	Never	3
Total		19

*Note:* Adapted from Geberhiwot et al. [8].

### 2.1 The Glut‐1CSS

The Glut1 Deficiency Syndrome Clinical Severity Score (Glut1‐CSS) was developed by the authors to provide a structured and reproducible tool for phenotypic stratification in patients with Glut1‐DS (Table 1). As no validated severity scale currently exists for Glut1‐DS, the structure of the Clinical Severity Scale proposed by Geberhiwot et al. for Niemann–Pick disease type C (NPC) was used as a conceptual framework due to its multidomain neurological assessment approach and ease of clinical applicability [8]. Although NPC and Glut1‐DS differ in pathophysiology and disease trajectory, both are chronic neurological disorders characterized by motor dysfunction, language impairment, seizures, and progressive neurological involvement. However, important adaptations were required to account for the distinct natural history and treatment responsiveness of Glut1‐DS.

The following methodological modifications were implemented:

- Domain selection: From the original NPC scale, only domains directly relevant to Glut1‐DS clinical expression were retained. Domains such as swallowing, manipulation, ophthalmoplegia, and neuropsychiatric subscales were excluded, as they are either less prevalent or not central to Glut1‐DS severity stratification in pediatric cohorts.
- Core domains defined for Glut1‐DS: Five essential domains were selected based on the most consistent clinical features reported in the literature—ambulation; language; seizures; age at independent gait acquisition; age at language acquisition.
- Ambulation modifications: The ambulation grading preserved the ordinal structure of the NPC model (0–5), but intermediate gradations were adapted to reflect the typical motor phenotype of Glut1‐DS, including—autonomous ataxic gait; outdoor assisted ambulation; indoor assisted ambulation; wheelchair dependency. This refinement allows differentiation between static motor impairment and more severe functional restriction, which is particularly relevant in treated patients.
- Language domain adjustments: The language domain was modified to better capture dysarthric phenotypes frequently observed in Glut1‐DS. Distinction was introduced between—mild dysarthria (fully intelligible); severe dysarthria (intelligible only to close family); nonverbal communication; absence of communication. This adaptation improves sensitivity to speech impairment severity.
- Seizure domain reconfiguration: Given the central role of epilepsy in Glut1‐DS, the seizure domain was reformulated to differentiate—no seizures; controlled with antiepileptic drugs; controlled with ketogenic diet; controlled with combined therapy; uncontrolled seizures. This distinction reflects therapeutic responsiveness, which is a key modifier of disease burden in Glut1‐DS and not addressed in the NPC model.
- Developmental milestones integration: Two additional domains: age at independent gait acquisition and age at language acquisition were incorporated. These are not part of the NPC scale but were included because developmental delay represents a defining and early feature of Glut1‐DS. Age thresholds were defined according to standard developmental milestones—before 18 months; between 18–24 months; after 24 months; never acquired.
- *Score structure and classification*: The final Glut1‐CSS ranges from 0 to 19 points. Based on distribution within our cohort and clinical interpretability, phenotypes were categorized as—mild: 0–7 points; moderate: 8–13 points; severe: 14–19 points. These cutoffs were empirically defined based on tertile distribution and clinical correlation.
- *Reproducibility*: This modified scale is not yet externally validated and should be considered an exploratory phenotypic stratification tool. However, it provides a structured approach for disease severity assessment in Glut1‐DS and may support future multicenter validation studies.

### 2.2 DNA Analysis

Genetic diagnostic approaches included single‐gene analysis through Sanger sequencing targeting *SLC2A1* (*n* = 2), as well as massive parallel sequencing (MPS)‐based gene panels that included *SLC2A1* and other genes associated with epilepsy and neurodevelopmental disorders (*n* = 16). Exome sequencing (ES) was performed in 17 cases. In 2 cases, the diagnosis was established through microarray analysis. In individuals with a known familial variant, targeted genotyping of the previously identified variant was conducted (*n* = 3).

Genetic variants were classified according to the American College of Medical Genetics and Genomics (ACMG) guidelines [9]. Variant frequency was analyzed using the Genome Aggregation Database (gnomAD). Variants not reported or reported at extremely low frequencies (< 0.01%) were considered more likely to be pathogenic in this rare disease cohort.

## 3 Results

### 3.1 Demographic Data

Forty patients from 36 families with a diagnosis of Glut1‐DS (Table 2). They came from 4 regions of Brazil (Southeast [*n* = 16], South [*n* = 11], Center‐West [*n* = 7], and Northeast [*n* = 6]).

**TABLE 2:** Summary of the Glut1‐DS patients included in this study (*n* = 40).

Pt	Family	Phenotype	Anticonvulsant drugs	Exon/intron	Variant	Diagnostic technique	Segregation in parents	ACMG classification
1	F1	Severe	No	E1	Arr[hg19] 1p34.2(43410251–43798716)x1, 10p15.3(2200633–2844138)x3 E1 deletion	Microarray	N	P
2	F2	Severe	No	E1 to 10	**Arr [hg19] 1p34.2p34.1(4183358805–44881988838)x1** **E1 to 10 deletion**	Microarray	N	P
3	F3	Moderate	No	E1 to 10	**E1 to 10 deletion**	Panel	N	P
4	F4	Mild	No	E2	c.101A>G; p.Asn34Ser	Panel	N	LP
5	F5	Moderate	Yes—Levetiracetam	E3	**c.227G>C; p.Gly76Ala**	ES	N	LP
6	F6	Moderate	Yes—Levetiracetam	E3	c.274delG; p.Arg92Glyfs*6	Panel	N	LP
7	F7	Mild	No	E3	c.236G>A; p.Gly79Asp	Familial variant testing	N	LP
8	F8	Moderate	Yes—Levetiracetam	E4	c.376C>T; p.Arg126Cys	ES	N	P
9	F9	Mild	No	E4	c.458G>A; p.Arg153His	ES	N	P
10	F10	Mild	No	E4	c.278G>A; p.Arg93Gln	Panel	N	P
11	F11	Severe	No	E4	c.316dup; p.Ser106Phefs*15	Panel	N	LP
12	F12	Mild	No	E4	c.418G>A; p.Vall40Met	ES	N	P
13	F13	Moderate	Yes—Levetiracetam, Lamotrigina,	E4	c.458G>A; p.Arg153His	ES	N	P
14	F14	Mild	No	E4	**c.464C>A; p.Ala155Asp**	Panel	N	LP
15	F15	Moderate	No	E5	c.557G>A; p.Trp186*	Panel	N	P
16	F16	Mild	No	E5	**c.524G>T; p.Gly175Val**	ES	N	LP
17	F17	Moderate	Yes—Levetiracetam	E5	c.635delG; p.Arg212Profs*17	Panel	N	P
18	F18	Moderate	No	E5	c.624_625insC; p.Glu209Argfs*28	ES	N	LP
19	F19	Mild	No	E6	c.855_856insTT; p.Gly286Leufs*55	ES	Y—De novo	LP
20	F20.1	Moderate	Yes—Lamotrigina, Clobazam	E6	c.835C>T; p.Gln279*	Panel	N	P
21	F20.2	Moderate	Yes—Lamotrigina, Clobazam	E6	c.835C>T; p.Gln279*	Panel	N	P
22	F21	Mild	Yes—Oxcarbazepina	E6	c.724C>T; p.Gln242*	ES	N	P
23	F22	Moderate	No	E7	c.884C>T; p.Thr295Met	Panel	N	P
24	F23	Mild	No	E7	c.884C>T; p.Thr295Met	Panel	N	P
25	F24	Moderate	Yes—Clobazan	E7	c.895G>T; p.Glu299*	Panel	N	LP
26	F25	Mild	No	E7	c.971C>T; p.Ser324Leu	Panel	N	P
27	F26	Moderate	No	E7	c.971C>T; p.Ser324Leu	ES	N	P
28	F27	Severe	Yes—Topiramato	E8	c.988C>T; p.Arg330*	ES	N	P
29	F28	Severe	No	E8	c.988C>T; p.Arg330*	ES	N	LP
30	F29 (sibling)	Severe	Yes—Levetiracetam	E8	c.997C>T; p.Arg333Trp	ES	N	P
31	F29 (sibling)	Severe	Yes—Levetiracetam	E8	c.997C>T; p.Arg333Trp	Panel	N	P
32	F30	Severe	Yes—Levetiracetam	E9	c.1075G>T; p.Glu359*	Panel	N	P
33	F31	Severe	Yes—Levetiracetam	E9	c.1189C>T; p.Gln397*	ES	N	P
34	F32 (son)	Mild	Yes—Valproato de Sódio	E9	**c.1147C>T; p.Pro383Ser**	Familial variant testing	Y—F	LP
35	F32 (father)	Mild	No	E9	**c.1147C>T; p.Pro383Ser**	Familial variant testing	N	LP
36	F33	Mild	No	E10	c.**1300 T>G; p.Phe434Val**	ES	N	LP
37	F34	Moderate	No	I2	**c.115‐2_115‐1delAG**	ES	N	LP
38	F35	Moderate	No	I3	c.275+1G>A	Single‐gene analysis	N	P
39	F36 (sibling)	Severe	Yes—Valproato de Sódio, Clobazam	I7	**c.972+4_972+5del**	ES	Y—M (mosaic)	VUS
40	F36 (sibling)	Severe	Yes—Valproato de Sódio, Clobazam	I7	**c.972+4_972+5del**	Single‐gene analysis	Y—M (mosaic)	VUS

*Note:* Bold type denotes novel variants (*n* = 15).

Abbreviations: ES, exome sequencing; F, father; LP, likely pathogenic; M, mother; P, pathogenic; VUS, variant of uncertain clinical significance.

### 3.2 Clinical Findings

Clinical data are shown in Table 2. Thirty‐two patients (80%) presented with the classic phenotype, and 8 (20%) with the nonclassic phenotype. Movement disorder was present in 29 patients (82%), speech delay in 34 (85%), motor delay in 26 (65%), and postnatal microcephaly in 4 patients (10%). Four families with recurrence of Glut1‐DS were identified, representing 20% of the sample. The GLUT1‐CSS scores of the patients included in the study are shown in Table 3.

**TABLE 3:** GLUT1 Clinical Severity Scale (GLUT1‐CSS) scores of the patients included in the study.

Patient	Family	Ambulation	Language	Seizure	Independent gait acquisition	Language acquisition	Total
1	F1	4	5	3	3	3	18
2	F2	5	4	1	3	3	16
3	F3	2	3	1	2	2	10
4	F4	1	0	0	0	0	1
5	F5	3	3	2	1	2	11
6	F6	3	3	2	2	2	12
7	F7	1	1	1	1	2	6
8	F8	2	5	1	2	3	13
9	F9	0	0	1	0	0	1
10	F10	0	0	1	0	0	1
11	F11	4	3	3	3	3	16
12	F12	1	1	0	0	0	2
13	F13	2	3	3	3	3	13
14	F14	0	0	1	0	0	1
15	F15	4	3	1	2	2	12
16	F16	1	2	1	1	1	6
17	F17	2	4	2	2	3	13
18	F18	1	2	1	2	2	8
19	F19	1	1	0	0	0	2
20	F20.1	2	2	3	2	2	13
21	F20.2	2	1	3	2	2	13
22	F21	2	1	2	0	2	7
23	F22	3	3	1	1	1	9
24	F23	1	1	2	1	1	6
25	F24	2	2	2	1	1	8
26	F25	2	2	1	0	2	7
27	F26	2	3	1	1	2	9
28	F27	4	6	1	2	3	16
29	F28	4	3	2	3	3	15
30	F29 (Sibling)	5	4	1	3	3	16
31	F29 (Sibling)	5	4	1	3	3	16
32	F30	4	5	2	2	2	15
33	F31	4	4	1	3	3	15
34	F32	2	2	1	2	0	7
34a	F32 (son)	0	2	0	1	1	4
35	F32	0	0	1	0	0	1
35a	F32 (father)	2	2	3	1	1	9
36	F33	4	3	1	2	2	12
37	F34	5	4	3	3	3	18
38	F35	5	4	3	3	3	18
39	F36 (sibling)	0	2	1	0	0	3
40	F36 (sibling)	2	2	1	2	1	8

### 3.3 Age and Severity Assessment

The median age at diagnosis was 5 years [IQR: 3.0–9.9]. There was a strong positive correlation between current age and age at diagnosis (*r* = 0.89, *p* < 0.0001). The Glut1‐CSS ranged from 1 to 18 points; the median score was 10.5 [IQR: 6.0–15.0]. Phenotypically, 14 patients (35%) belonged to the mild subgroup, 15 (37.5%) to the moderate subgroup, and 11 (27.5%) to the severe subgroup.

### 3.4 Ketogenic Diet and Severity Assessment

Thirty‐four patients were on a ketogenic diet (85%). The median time on the ketogenic diet was 2.95 years [IQR: 0.2–18.8]. Twenty‐seven were on a classic ketogenic diet (79.4%) and 7 on modified Atkins diets (20.6%). All patients on a modified Atkins diet were over 8 years of age (median, 12.2 years [IQR: 0.85–23.0]). In this group, the median Glut1‐CSS score was 13 [IQR: 10–14.5]. The ratio of the classic diet was between 1:1 and 3:1, with no patients having a ratio of 4:1. In 11 patients (31.4%), seizure control was achieved exclusively with the ketogenic diet. Other reported effects included improvement in gait, speech, and ataxia. The reported adverse effects were transient and occurred at the beginning of treatment. The most common were constipation (28.5%), irritability (8.5%), and nausea (5.7%).

Among the 40 patients in the cohort, 37 (92.5%) experienced seizures. The median age at seizure onset was 0.2 years [IQR: 0.1–0.6]. Of these, 34 (91.9%) were on a ketogenic diet as part of their treatment for seizure control, and 17 patients (45.9%) received antiepileptic drugs in their therapeutic regimen. Overall, 21 patients (56.7%) relied exclusively on a ketogenic diet for seizure management, while 13 patients (35.1%) utilized a combination of a ketogenic diet and antiepileptic drugs. Three patients (8.1%) with a history of seizures managed their condition exclusively with antiepileptic drugs. Among the remaining 3 patients, one (2.2%) had no history of seizures and did not utilize a ketogenic diet or antiepileptic drugs, while one patient (0.3%) had a history of seizures but maintained seizure control without the use of the ketogenic diet or antiepileptic drugs.

Patients using only the ketogenic diet exhibited a milder clinical presentation compared to those on antiepileptic drugs or a combination of ketogenic diet and antiepileptic drugs. The median Glut1‐CSS was 7 [IQR: 2–10] for the first group, compared to 13 [IQR: 11.25–15.75] for the latter 2 groups.

Among the 18 patients with seizures who were managed exclusively with the classic ketogenic diet, 10 (55.5%) were on a 3:1 ratio, 2 (11.1%) on a 2.5:1 ratio, 5 (27.7%) on a 2:1 ratio, and 1 (5.5%) on a 1:1 ratio. Conversely, among the 9 patients with a history of seizures who were managed with the classic ketogenic diet in combination with antiepileptic drugs, only 2 (22.2%) were on a 3:1 ratio, 4 (44.4%) on a 2:1 ratio, and 3 (33.3%) on a 1:1 ratio.

Among the 17 patients receiving antiepileptic drugs for seizure control, 12 (70.5%) were on monotherapy, while 5 (29.5%) used a combination of 2 medications. The most common antiepileptic drug was levetiracetam, prescribed in 53% of cases.

### 3.5 Association Between Clinical Variables and Disease Severity

**FIGURE 1:**
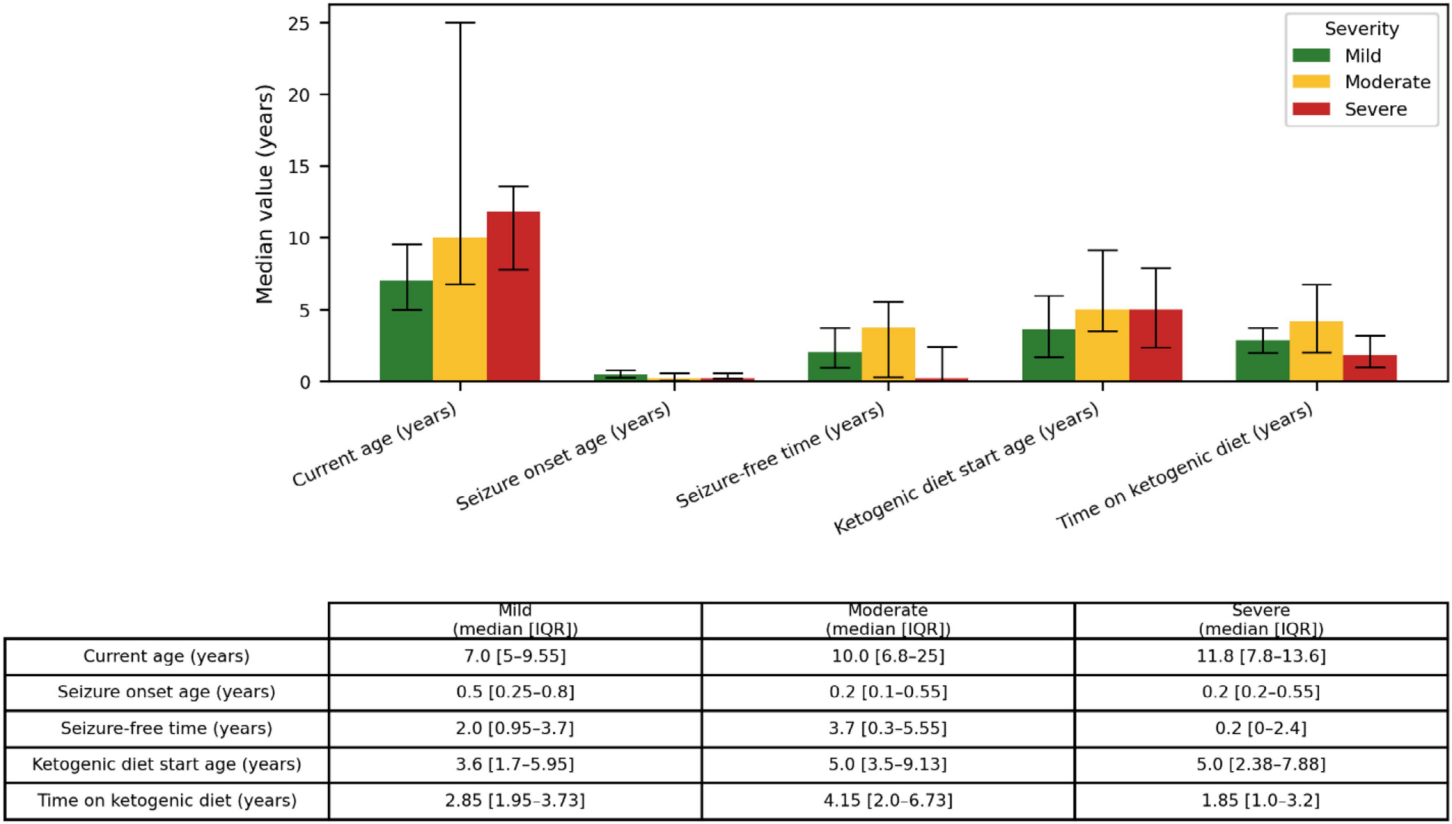
Clinical variables and disease severity based on Glut1‐CSS. Legend: Median values (IQR) for current age, age at seizure onset, seizure‐free interval (from the initiation of ketogenic diet treatment in individuals who had uncontrolled seizures at treatment onset), age at initiation of ketogenic diet, and time on ketogenic diet for patients with mild, moderate, and severe Glut1‐CSS. Disease severity was determined using the Glut1‐CSS, which ranges from 0 to 19 and integrates five domains: Ambulation, language, seizure control, age at independent gait acquisition, and age at language acquisition. The total Glut1‐CSS score defines severity as mild (0–7 points), moderate (8–13 points), or severe (14–19 points). Scores are assigned as follows: Ambulation (0–5): Normal (0), clumsy (1), autonomous ataxic gait (2), outdoor assisted (3), indoor assisted (4), wheelchair‐bound (5); Language (0–5): Normal (0), delayed acquisition (1), mild dysarthria (2), severe dysarthria (3), nonverbal communication (4), absence of communication (5); Seizures (0–3): Absent (0), controlled by antiepileptic drugs or ketogenic diet (1), controlled by both (2), uncontrolled (3); Independent gait acquisition (0–3): Before 18 months (0), between 18–24 months (1), after 2 years (2), never (3); and Language acquisition (0–3): Before 18 months (0), between 18–24 months (1), after 2 years (2), never (3).

Descriptive analysis of clinical variables, based on the Glut1‐CSS, revealed a trend of increasing values with greater phenotypic severity (Figure 1). Patient age was higher in the moderate and severe groups compared to the mild group (median ages: 7.0 [IQR: 5.18–9.73], 10.0 [IQR: 6.8–25.0], and 11.8 years [IQR: 7.8–13.6], respectively). Seizures tended to begin earlier in patients with more severe phenotypes (median ages: 0.5 years [IQR: 0.25–0.8] in the mild group and 0.2 years in both the moderate [IQR: 0.1–0.55] and severe [IQR: 0.2–0.55] groups).

The seizure‐free interval also differed among groups, with longer duration in the mild and moderate subgroups compared to the severe group (medians: 2 [IQR: 0.95–3.7], 3.7 [IQR: 0.3–5.55], and 0.2 years [IQR: 0.0–2.4], respectively). These findings suggest more limited seizure control over time in individuals with more severe clinical presentations.

The age at initiation of the ketogenic diet was slightly older among patients with more severe phenotypes (medians: 3.6 [IQR: 1.7–5.95], 5.0 [IQR: 3.5–9.13], and 5.0 [IQR: 2.38–7.88] years for the mild, moderate, and severe groups, respectively). Moreover, the total time on the ketogenic diet was shorter in the severe group (median durations: 2.85 years [IQR: 1.95–3.73] for mild, 4.15 years [IQR: 2.0–6.73] for moderate, and 1.85 years [IQR: 1.0–3.2] for severe).

### 3.6 Genetic Findings

DNA testing was performed in all patients. Thirty‐one variants were found in heterozygosity: 15 (48.3%) missense, 6 (19.3%) nonsense, 5 (16.1%) frameshift, 2 (6.4%) deletions, 2 (6.4%) intronic, and one (3.2%) indel. Fifteen variants (37.5%) were novel (Table 2). Three of these variants were classified as pathogenic, 11 as probably pathogenic, and one as a variant of unknown significance (VUS) (c.972+4_972+5del). This variant falls in intron 7 of the *SLC2A1* gene, is not present in population databases, and has not been reported in previous literature in individuals with Glut1‐DS. Algorithms developed to predict the effect of sequence changes on RNA splicing suggest that this variant may disrupt the consensus splice site, but this prediction has not been confirmed by published transcriptional studies. This variant was identified in two siblings from Family F36, a 15‐year‐old girl and her 8‐year‐old brother, born to a healthy, non‐consanguineous couple after uneventful pregnancies and deliveries, with normal anthropometric parameters at birth. The girl had her first seizure at 3 months of age, and the boy at 26 days. Both have severe neuropsychomotor developmental delay and are currently nonambulatory and nonverbal. Cerebrospinal fluid analysis revealed hypoglycorrhachia in the boy. Segregation analysis showed maternal mosaicism for the variant, and the mother remains asymptomatic.

### 3.7 Genotype/Phenotype Correlation

Of those patients with a nonclassical phenotype, 7 (87%) had missense variants and only one (13%) had a frameshift variant. Among those with a classic phenotype, 13 (41%) had missense variants, 8 (25%) nonsense variants, 4 (13%) frameshift variants, 3 (9%) deletions, 3 (9%) intronic variants, and 1 (3%) indel.

Patients with missense variants more frequently exhibited a mild phenotype (60% mild, 30% moderate, and 20% severe), compared to patients with frameshift variants (20% mild, 60% moderate, 20% severe), nonsense (12.5% mild, 37.5% moderate, 50% severe), deletions (0% mild, 33.3% moderate, 66.6% severe), and intronics (0% mild, 33.3% moderate, 66.6% severe).

## 4 Discussion

This study provides the first comprehensive evaluation of patients with Glut1‐DS in the Brazilian population, contributing significantly to the understanding of this condition in a population context that has been underexplored. We report 15 novel variants in the *SLC2A1* gene, representing an additional 5% to the total number of previously known variants. These findings expand the genotypic spectrum of Glut1‐DS and highlight the importance of ongoing genetic investigations to identify new variants, which can contribute to a better understanding of the disease's molecular basis and its phenotypic variability. In addition, we propose a new scoring system for the classification of disease severity, the Glut1‐CSS, which appears to be useful for distinguishing patients with mild, moderate, and severe forms of the disorder.

Our results confirm the phenotypic variability described in the literature, highlighting the predominance of the classic phenotype. The proportion found (80%) is consistent with previous findings, such as those of Leen et al., who observed a classic phenotype in approximately 84% of patients [10]. Hully et al. evaluated 58 patients with Glut1‐DS and, as in our study, 87.5% presented with the classic phenotype [11]. Similarly, Wang et al. reported that the nonclassic phenotype was present in around 15%–20% of patients with Glut1‐DS [12].

The correlation found between current age and age at diagnosis likely reflects a cohort effect: older patients had onset of symptoms at times when knowledge of Glut1‐DS was more limited, clinical suspicion was low, and molecular testing or CSF analysis was not routinely available, resulting in delayed recognition and a longer diagnostic journey. In contrast, younger patients have benefited from recent advances in genomic technologies, broader availability of targeted panels and ES, and increased awareness of the syndrome among pediatric neurologists and geneticists, enabling earlier diagnosis.

The correlation between phenotype severity and the age of seizure onset was evident in our cohort, with earlier onset associated with more severe manifestations. This finding aligns with the observations of Leen et al., who highlighted the negative impact of chronic cerebral glucose deprivation during critical phases of neurocognitive development, especially in patients who were not treated during the early years of life. These data underscore the importance of early diagnostic strategies, including genomic studies and CSF analysis, which have proven effective in identifying hypoglycorrhachia as a diagnostic marker in over 90% of cases [10].

Another relevant finding was the predominance of missense variants (48.3%), followed by nonsense and frameshift variants, consistent with previous reports. In our cohort, variant type was significantly associated with phenotypic severity: missense variants were more frequently observed in patients with mild phenotypes, whereas truncating variants were predominantly associated with severe clinical presentations. This pattern aligns with prior studies demonstrating that variants leading to more substantial loss of GLUT1 function are associated with more pronounced neurological impairment, including intellectual disability and motor dysfunction [10, 11, 12].

Functional studies have further supported this relationship, showing that nonsense variants exhibit a greater negative impact on transporter activity compared to missense variants [13]. However, phenotypic variability was also observed in our cohort, including a patient carrying a frameshift variant who presented with a mild phenotype. Such exceptions, also described previously, highlight the complexity of genotype–phenotype correlations in Glut1‐DS and suggest that additional modifying factors may influence clinical expressivity [13, 14, 15].

Although Glut1‐DS is predominantly associated with de novo pathogenic variants in *SLC2A1*, familial transmission has been increasingly recognized. In our cohort, recurrence was observed in 20% of cases, a proportion higher than traditionally reported. This finding may reflect systematic family screening and the identification of mildly affected or asymptomatic parental carriers, including cases of somatic mosaicism, rather than a truly elevated recurrence risk. No consanguinity was identified in these families, supporting autosomal dominant inheritance rather than recessive transmission, and parental ages did not demonstrate a consistent pattern suggestive of an advanced paternal age effect. The observation of vertical transmission, including from oligosymptomatic or asymptomatic parents, reinforces the need for systematic molecular testing in parents and first‐degree relatives of affected individuals. Incomplete penetrance and variable expressivity, well documented in Glut1‐DS, limit the ability to reliably predict clinical severity based solely on the identified variant [5, 16, 17, 18, 19]. Together, these findings highlight that familial recurrence, although less frequent than de novo occurrence, may be underrecognized and warrants careful clinical and molecular evaluation of relatives, with direct implications for recurrence risk assessment and genetic counseling.

Takahashi et al. described a familial case of Glut1‐DS in which somatic mosaicism for a truncating *SLC2A1* variant (c.1303dupA, p.Ile435Asnfs*20) was identified in the mother, who presented only mild paroxysmal nonkinesigenic dyskinesia, whereas her son, carrying the variant in heterozygous state, exhibited a classic severe phenotype with early‐onset seizures and developmental delay [20]. The maternal variant allele fraction ranged from 28% to 34% across analyzed tissues, compared to approximately 50% in the affected child, illustrating how mosaicism level may influence clinical expressivity. These findings highlight that mosaicism should be considered in individuals with suggestive features of Glut1‐DS, such as early‐onset epilepsy, movement disorders, hypoglycorrhachia, or favorable response to ketogenic diet, particularly when conventional sequencing fails to detect a pathogenic heterozygous variant. Because low‐level mosaicism may escape detection by standard Sanger sequencing, high‐depth next‐generation sequencing with variant allele fraction analysis and, when appropriate, testing of multiple tissues may be required. In our cohort, the identification of an asymptomatic mother carrying a pathogenic variant further supports the possibility that mosaicism or additional modifying mechanisms may contribute to phenotypic variability, underscoring its relevance for diagnostic accuracy and genetic counseling.

The acknowledgment that Glut1‐DS demonstrates incomplete penetrance and highly variable expressivity has important implications for family genetic counseling. Variant segregation analysis should always be performed in the parents of affected individuals, even if they are asymptomatic, as part of a comprehensive strategy for diagnosis and management of the syndrome.

The main limitation of our study concerns the retrospective design, which may have introduced registration bias and hindered the standardization of clinical assessments. Future studies should focus on expanding genomic analysis and exploring the modifying factors that may influence phenotypic expression, with the aim of optimizing clinical management and improving patient outcomes.

## 5 Conclusions

This is the first study to examine the natural history of Glut1‐DS in the Brazilian population and to apply the Glut1‐CSS, providing valuable insights into a diverse and underrepresented setting. Our findings confirm the predominance of the classic phenotype, characterized by early‐onset seizures, developmental delay, and movement disorders, while also highlighting substantial phenotypic variability. The identification of 15 novel variants and the detection of mosaicism in familial cases underscore the genetic complexity of the syndrome and reinforce the need for comprehensive molecular investigation, including high‐depth next‐generation sequencing and parental testing when clinical suspicion persists despite negative initial results.

Although ketogenic diet therapy was associated with clinical improvement in seizure frequency and neurodevelopmental trajectory, particularly when introduced early, complete seizure freedom was achieved in only a subset of patients. This finding suggests that, while the ketogenic diet remains the cornerstone of treatment, therapeutic response may vary, and early diagnosis plays a critical role in optimizing outcomes rather than guaranteeing seizure remission.

The observed correlation between greater clinical severity and delayed diagnosis further emphasizes the importance of increasing awareness among healthcare professionals and expanding access to specialized genetic testing. Given the presence of familial transmission, incomplete penetrance, and mosaicism in our cohort, genetic evaluation in suspected Glut1‐DS should include not only sequencing of the *SLC2A1* gene but also consideration of mosaic variants and systematic parental analysis to ensure accurate diagnosis, appropriate counseling, and informed recurrence risk assessment. Addressing regional disparities in diagnostic access remains essential to reduce underdiagnosis and improve long‐term outcomes for individuals with Glut1‐DS.

## Author Contributions

**Lívia Maria Ferreira Sobrinho:** conception, data acquisition, data analysis and interpretation, technical procedures, manuscript writing. **Juliana Cristine Fontana:** data analysis and interpretation, technical procedures, critical review. **Claudia F. Lorea:** data acquisition, critical review. **Helio van der Linden Júnior:** data acquisition, critical review. **Andréa C. S. Leite:** data acquisition, critical review. **Rayana Elias Maia:** data acquisition, critical review. **Lilia Farret:** data acquisition, data analysis and interpretation, technical procedures, critical review. **Carolina F. Moura de Souza:** data acquisition, technical procedures, critical review. **Soraia Poloni:** data acquisition, data analysis and interpretation, technical procedures, critical review. **Fabiano O. Poswar:** data acquisition, data analysis and interpretation, technical procedures, data analysis and interpretation, technical procedures, critical review. **Ida Vanessa D. Schwartz:** data acquisition, data analysis and interpretation, technical procedures, data analysis and interpretation, technical procedures, critical review, final approval. **Fernanda Sperb‐Ludwig:** data acquisition, data analysis and interpretation, technical procedures, critical review.

## Funding

This work was supported by Financiamento e Incentivo à Pesquisa (Fipe/HCPA).

## Ethics Statement

All procedures followed were in accordance with the ethical standards of the responsible committee on human experimentation (institutional and national) and with the Helsinki Declaration of 1975, as revised in 2000. This study was approved by Research Ethics Committee number 5327 of Hospital de Clínicas de Porto Alegre (Certificate of Submission for Ethical Approval: 65118222.5.0000.5327).

## Consent

Informed consent was obtained from the legal guardians of the patients included in the study.

## Conflicts of Interest

The authors declare no conflicts of interest.

## Data Availability

The data that support the findings of this study are available from the corresponding author upon reasonable request.
